# Association of knee atypical lesions with foot ulcers and a peak of kappa IgM monoclonal immunoglobulin

**DOI:** 10.1016/j.jdcr.2022.12.016

**Published:** 2023-01-11

**Authors:** Marine Robert, Marie-Anne Couturier, Nicolas Ortonne, Glen Le Flahec, Céline Bryer-Le Breton, Flavien Huet, Laurent Misery

**Affiliations:** a Department of Dermatology, Brest University Hospital, Brest, France; b Department of Hematology, Brest University Hospital, Brest, France; c Department of Pathology, Henri-Mondor Hospital, AP-HP, Créteil, France; d Department of Pathology, Brest University Hospital, Brest, France; e Department of Neurology, Brest University Hospital, Brest, France

**Keywords:** cutaneous macroglobulinosis, plantar disease, Waldenström’s disease, CM, cutaneous macroglobulinosis

## Presentation

**Fig 1:**
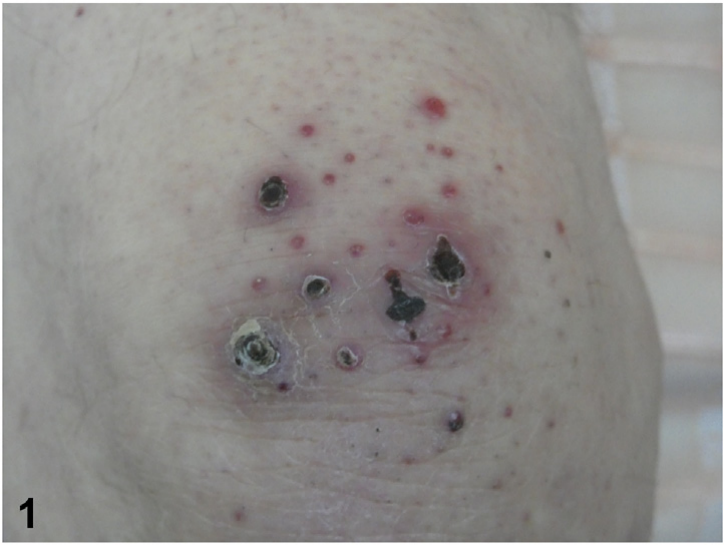

**Fig 2:**
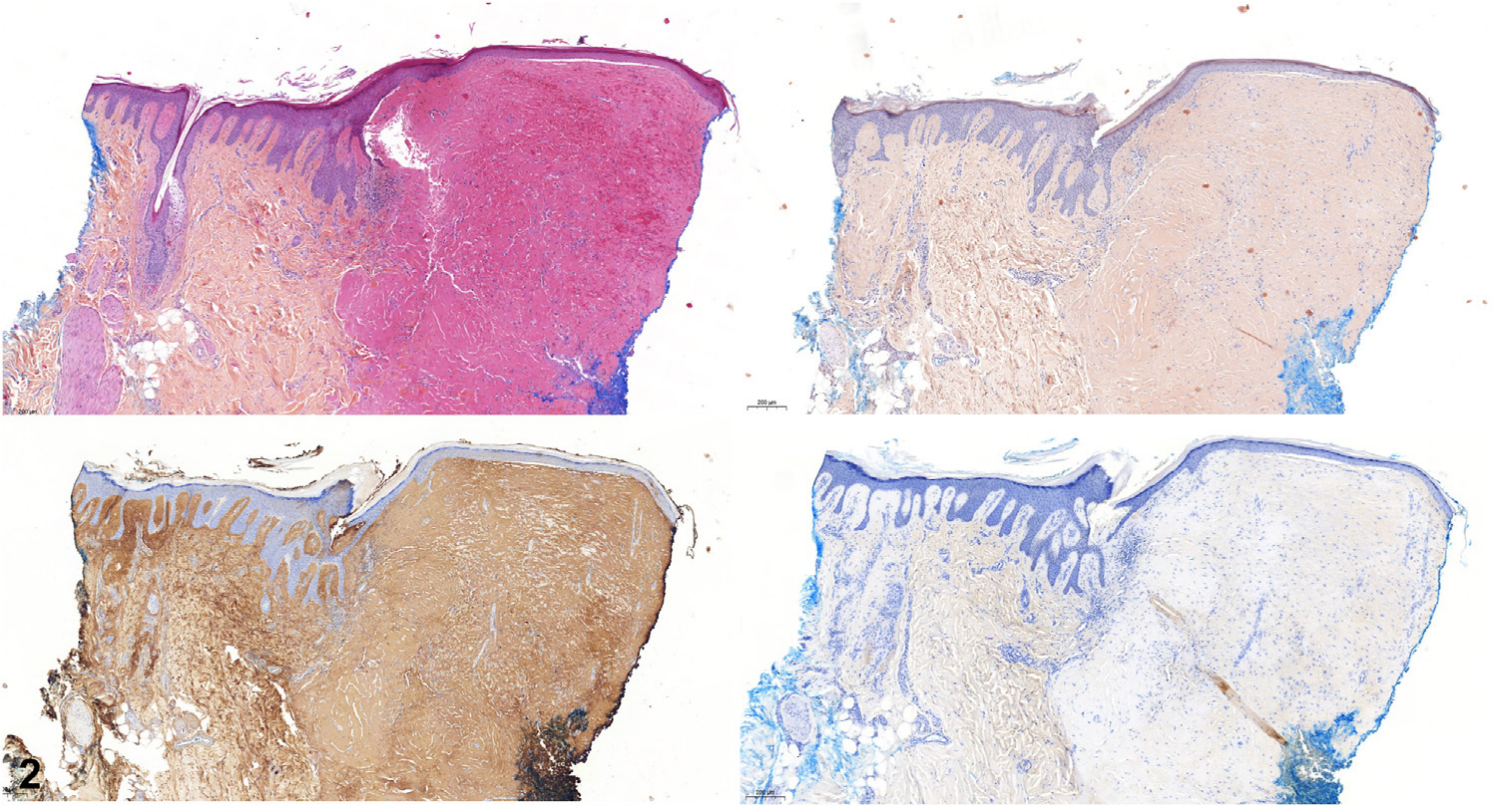

An 84-year-old man presented with small erythematous, necrotic papules on the knees (Fig 1) and the dorsal side of hands, associated with 2 plantar ulcers and paraesthesias. Pancytopenia and a peak of kappa IgM monoclonal immunoglobulin (28.3 g/L), with free kappa light chains, were noted. A Bence-Jones kappa-like proteinuria was present. Bone marrow aspirate analysis revealed a B-cell infiltration (45% of CD19+ cells) with intermediate kappa immunoglubulins (92%). Immunohistochemical analysis of knee lesion revealed large deposits of amyloid-like substance that was immunoreactive for anti-kappa antibody (Fig 2). Computed tomography scan showed osteoarthritis of the left fifth metatarsophalangeal joint. Electroneuromyography revealed axonal sensorimotor polyneuropathy.

**Question 1: What is the diagnosis ofcutaneous** **lesions on the knees?**

A. Purpura secondary to thrombocytopenia
B. Wounds secondary to trauma
C. Skin infection
D. Amyloidosis
E. Macroglobulinosis

**Answers:**

A. Purpura secondary to thrombocytopenia – Incorrect. These lesions are rather necrotic and no other purpuric lesions were observed. Thrombocytopenia was not severe (platelet count: 78 × 10^9^/L).
B. Wounds secondary to trauma – Incorrect. No trauma was reported.
C. Skin infection – Incorrect. There was no erythematous border or pus.
D. Cutaneous amyloidosis – Incorrect. No apple-green birefringence with Congo red staining on polarized light microscopy was observed. Cutaneous symptoms of amyloidosis are a peculiar form of *purpura* in a periorbital distribution (“raccoon eyes”), waxy thickening of the skin, easy bruising, and *subcutaneous nodules* or plaques, which are very different lesions from those of this patient. Hence, the diagnosis of cutaneous amyloidosis can be excluded clinically.
E. Cutaneous macroglobulinosis (CM) – Correct. CM is characterized by the deposition of eosinophilic, immunoglobulin-derived material in the dermis. It typically presents as pink or skin-colored papules favoring the extensor surfaces of the extremities.^1^ It is rare and only some small case series have been reported.^2^

**Question 2: What is the hematological diagnosis?**

A. Lymphoid leukemia
B. Myeloma
C. Cutaneous lymphoma
D. Monoclonal gammopathy of undermined significance
E. Waldenström’s disease

**Answers:**

A. Lymphoid leukemia – Incorrect. There was no lymphoproliferative disorder in the blood.
B. Myeloma – Incorrect. The most common type of myeloma is IgG kappa (rare cases with IgA or even IgD or IgE). A monoclonal peak of kappa IgM is typical of Waldenström’s disease while IgM myeloma is extremely rare.^3^
C. Cutaneous lymphoma – Incorrect. There were no lymphoma cells in the skin.
D. Monoclonal gammopathy of undermined significance – Incorrect. The Ig level is lower than in myeloma and there are no clinical symptoms
E. Waldenström’s disease – Correct. Waldenström’s disease is a rare subtype of lymphoplasmacytic lymphoma characterized by monoclonal proliferation of lymphoplasmacytes in the bone marrow, lymph nodes, and spleen. Increased levels of circulating IgM monoclonal antibodies lead to their deposition in the skin and other organs. Cutaneous manifestations are rare and can be classified into specific and non-specific findings.^4^^,^^5^ Blood samples or biopsy of bone marrow, lymph nodes, or other organs likely to be infiltrated may be analyzed for proteins on tumor cells to distinguish Waldenström’s macroglobulinemia from other types of B-cell lymphomas. Flow cytometry immunophenotyping is essential for establishing the presence of a monotypic B-cell disease component but also helping to exclude the possibility of chronic lymphocytic leukemia/small lymphocytic lymphoma.^3^

**Question 3: What is CM?**

A. An equivalent of amyloidosis in Waldenström’s disease
B. An indirect consequence of the deposition of immunoglobulins
C. A specific manifestation of Waldenström’s disease
D. A frequent condition
E. A predictive factor in all cases

**Answers:**

A. An equivalent of amyloidosis in Waldenström’s disease – Incorrect. Amyloidosis refers to the deposition of fibrils within extracellular tissues of light chains in a ß-pleated sheet configuration.^1^
B. An indirect consequence of the deposition of immunoglobulins – Incorrect. IgM is a polymer characterized by multiple immunoglobulins linked together by strong *covalent bonds*, giving rise to pentamers. The combination of increased *vascular permeability*, high serum concentration, and presence of *specific antigens* in the skin may result in its deposition within the *dermis*.^1^ Hence CM is a direct consequence of the deposition of immunoglobulins.
C. A specific manifestation of Waldenström’s disease – Correct. Non-specific findings are attributed to hyperviscosity or cryoglobulinemia, whereas specific manifestations are related to neoplastic B-cell infiltrates and monoclonal IgM deposition in the skin.^4^^,^^5^ This case is notable because Waldenström’s disease was revealed by both non-specific (neuropathic ulcers) and specific (CM) skin manifestations. The occurrence of the neuropathy is thought to be related to the accumulation of IgM in myelin sheaths.^3^
D. A frequent condition – Incorrect. It is very rare and there are only some case reports in the literature.^1^^,^^4^^,^^5^
E. A predictive factor in all cases – Incorrect. Patients can develop CM before, concurrent with, or – as in our reported case – after diagnosis of the underlying lymphoplasmacytic lymphoma. Hence, CM can predict a latent plasma cell dyscrasia before any other clinical or pathologic evidence^5^ but not in all cases.

## Conflicts of interest

None disclosed.

## References

[1] Alegría-Landa V., Cerroni L., Kutzner H., Requena L. (2017). Paraprotein deposits in the skin. J Am Acad Dermatol.

[2] Hassab-El-Naby H.M., El-Khalawany M., Rageh M.A. (2020). Cutaneous macroglobulinosis with Waldenstrom macroglobulinemia. JAAD Case Rep.

[3] Ansell S.M., Kyle R.A., Reeder C.B. (2010). Diagnosis and management of Waldenström macroglobulinemia: Mayo stratification of macroglobulinemia and risk-adapted therapy (mSMART) guidelines. Mayo Clin Proc.

[4] Libow L.F., Mawhinney J.P., Bessinger G.T. (2001). Cutaneous Waldenstrom's macroglobulinemia: report of a case and overview of the spectrum of cutaneous disease. J Am Acad Dermatol.

[5] Yokote T., Akioka T., Oka S. (2006). Cutaneous infiltration with Waldenström macroglobulinemia. Leuk Res.

